# Resistance profiles of fastidious oral anaerobes and other periodontal species by multipoint inoculator: an *in vitro* study

**DOI:** 10.3389/fdmed.2026.1753219

**Published:** 2026-03-26

**Authors:** Alexandra Wolf, Alexander Indra, Florian Heger, Julia Reichl, Andreas Moritz, Apostolos Georgopoulos

**Affiliations:** 1 Core Facility for Oral Microbiology and Hygiene, University Dental Clinic, Vienna, Austria; 2 University Dental Clinic Vienna, Vienna, Austria; 3 Austrian Agency for Health and Food Safety (AGES), Vienna, Austria; 4 Medical University of Vienna, Vienna, Austria

**Keywords:** anaerobic bacteria, antibiotic resistance, biofilm, MIC testing, multipointinoculator, periodontal disease

## Abstract

Periodontal disease is one of the most prevalent chronic infections worldwide. Associations with systemic conditions, including endocarditis, type 2 diabetes mellitus, cardiovascular disease, and tumor progression, have been extensively investigated in recent studies. Furthermore, it involves pathogens that are often anaerobic, biofilm-associated, and difficult to treat owing to inherent resistance mechanisms and the protective nature of biofilms. As dentists prescribe a substantial proportion of antibiotics and antibiotic resistance is increasing globally, innovative diagnostic methods are urgently needed to ensure effective and sustainable antibiotic stewardship. In this study, anaerobic stock isolates from 30 to 35 frozen samples per genera or species were subjected to minimum inhibitory concentration testing. A multipoint inoculator was used to seed agar plates containing serial antibiotic dilutions, allowing assessment of five commonly used antibiotics: amoxicillin, amoxicillin/clavulanic acid, imipenem, clarithromycin, and levofloxacin. This enabled simultaneous testing of multiple strains under strictly anaerobic conditions, reducing the handling time and material consumption. Among the tested oral pathogens, high resistance levels were observed for clarithromycin. Amoxicillin (±clavulanic acid) remains the most reliable first-line option, whereas levofloxacin may serve as an alternative for certain gram negative anaerobes, when standard treatment protocols are unsuccessful. Mixed-inoculum experiments further showed altered antibiotic responses under biofilm-like conditions. The multipoint inoculator enables reliable testing of oxygen-sensitive anaerobes, including mixed biofilms.

**Clinical relevance:**

The presented method enables dental practitioners and laboratories to obtain resistance data from subgingival pathogens, especially in low- and middle-income countries or remote regions where access to centralized microbiology laboratories is limited. This benefits the wider healthcare system by supporting sustainable antibiotic stewardship through reliable, cost-effective resistance testing and the patients by facilitating the selection of an effective targeted agent, particularly in complex cases where multiple empirical antibiotic therapies have failed.

## 1 Introduction

### 1.1 Periodontal disease

Periodontal disease is one of the most common chronic infections worldwide, affecting a considerable proportion of the adult population (1). Its onset and progression are driven by a complex interaction of microorganisms in the oral cavity (2). Periodontal disease causes swelling, pain, and eventually tooth loss, whereas its systemic impact extends far beyond the oral cavity. Periodontal pathogens may persist locally or disseminate systemically (2). Certain species, including *Porphyromonas gingivalis* (*P. gingivalis.*) and *Fusobacterium nucleatum* (*F. nucleatum.*), have been implicated in Alzheimer's disease (3, 4) and tumor progression (5, 6). Furthermore, chronic oral inflammation has been associated with type 2 diabetes mellitus (7, 8), cardiovascular disease, inflammatory bowel disease, and rheumatoid arthritis (9). Untreated infections exacerbate chronic conditions, increase healthcare costs, and reduce healthy life years. Gram-positive bacteria such as Streptococcus spp. are among the most important early colonizers of the oral cavity. They are characterized by strong adhesion, rapid adaptability, and a central role in the development of caries and endocarditis (10), especially representatives of the *viridans streptococcus group* such as *S. mutans* and *S. sanguinis*. Members of the *Peptostreptococcaceae* family, especially *Parvimonas micra (P. micra*), are associated with periodontitis; they produce proteolytic enzymes and volatile sulfur compounds that contribute to tissue destruction and oral halitosis (11). *Actinomycetes* spp. are early colonizers, crucial in biofilm maturation. Although generally commensal, some species (e.g., *A. naeslundii*) can induce gingival inflammation and stimulate the growth of periodontal pathogens (10). Bacteroidaceae represent a heterogeneous group of gram-negative anaerobic rods, with *P.gingivalis*, *Prevotella* spp, *Tannerella forsythia (T. forsythia)*, and *F. nuculatum* being particularly relevant in periodontology. *P. gingivalis*, a key pathogen in periodontitis, thrives in subgingival pockets and evades host defense via proteolytic metabolism, capsule formation, and immune cell suppression (11). *Prevotella* spp., particularly *P. intermedia*, contribute to gingivitis and periodontitis via fimbriae-mediated adhesion, cytokine induction, and protease activity, whereas *T. forsythia* contributes to tissue invasion and apoptosis of lymphocytes in conjunction with *P. gingivalis. T. forsythia* is hard to cultivate in the absence of other bacteria, such as *P. gingivalis* or *F. nucleatum* due to its absence of common peptide glycan biosynthesis genes (12). *F. nucleatum* acts as a bridging organism within biofilms, enabling co-aggregation of pathogenic species and linking early to late colonizers, thereby playing a pivotal role in periodontal dysbiosis and systemic associations, such as cancer (11). *Veillonella* spp. use lactate from *Streptococcus* spp. and *Actinomyces* spp., contributing to biofilm stability and metabolic networks within the oral microbiome (13). Anaerobic gram-negative pathogens, such as *Prevotella* spp*.*, *Porphyromonas* spp*.*, and *Fusobacterium nucleatum*, play a central role in gingivitis-to-periodontitis transition. Biofilm-mediated protection enhances resistance against host defenses and antimicrobial agents (14).

**Figure 1:**
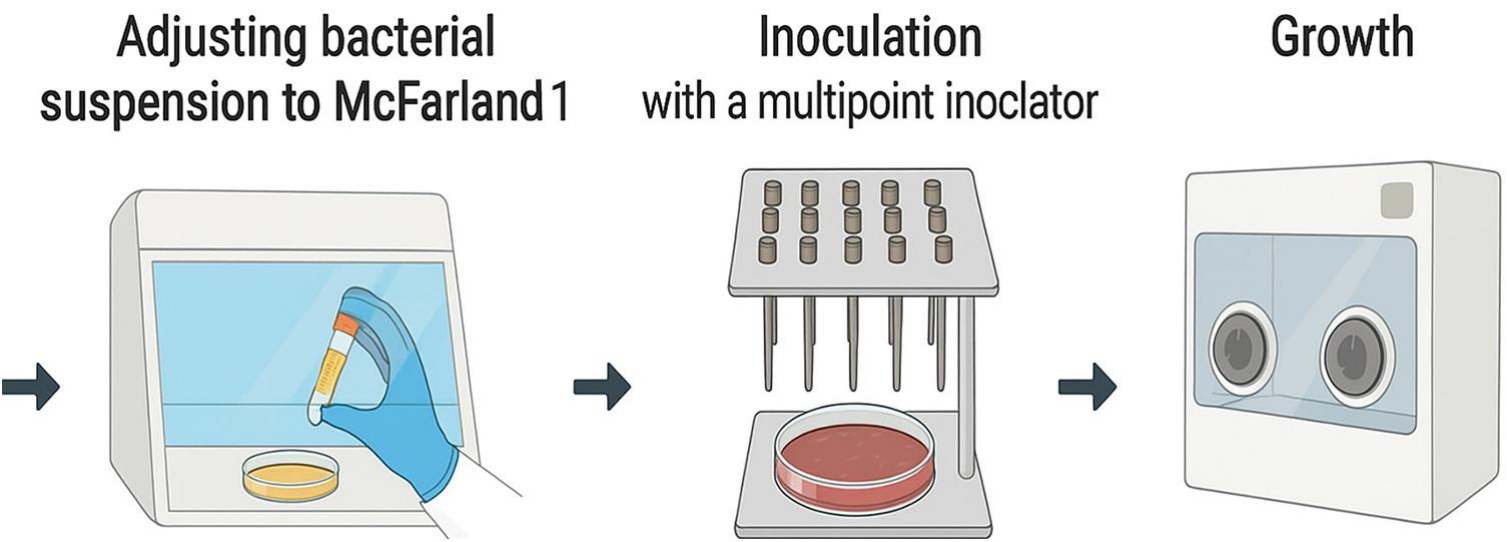
Culture preparation (1 McF for anaerobe testing), inoculation using a multipoint inoculator and cultivation in an anaerobe chamber an anaerobic chamber 80% N_2_, 15% CO_2_, and 5% H_2_. 37°C for 72 h to 7 days.

**Figure 2:**
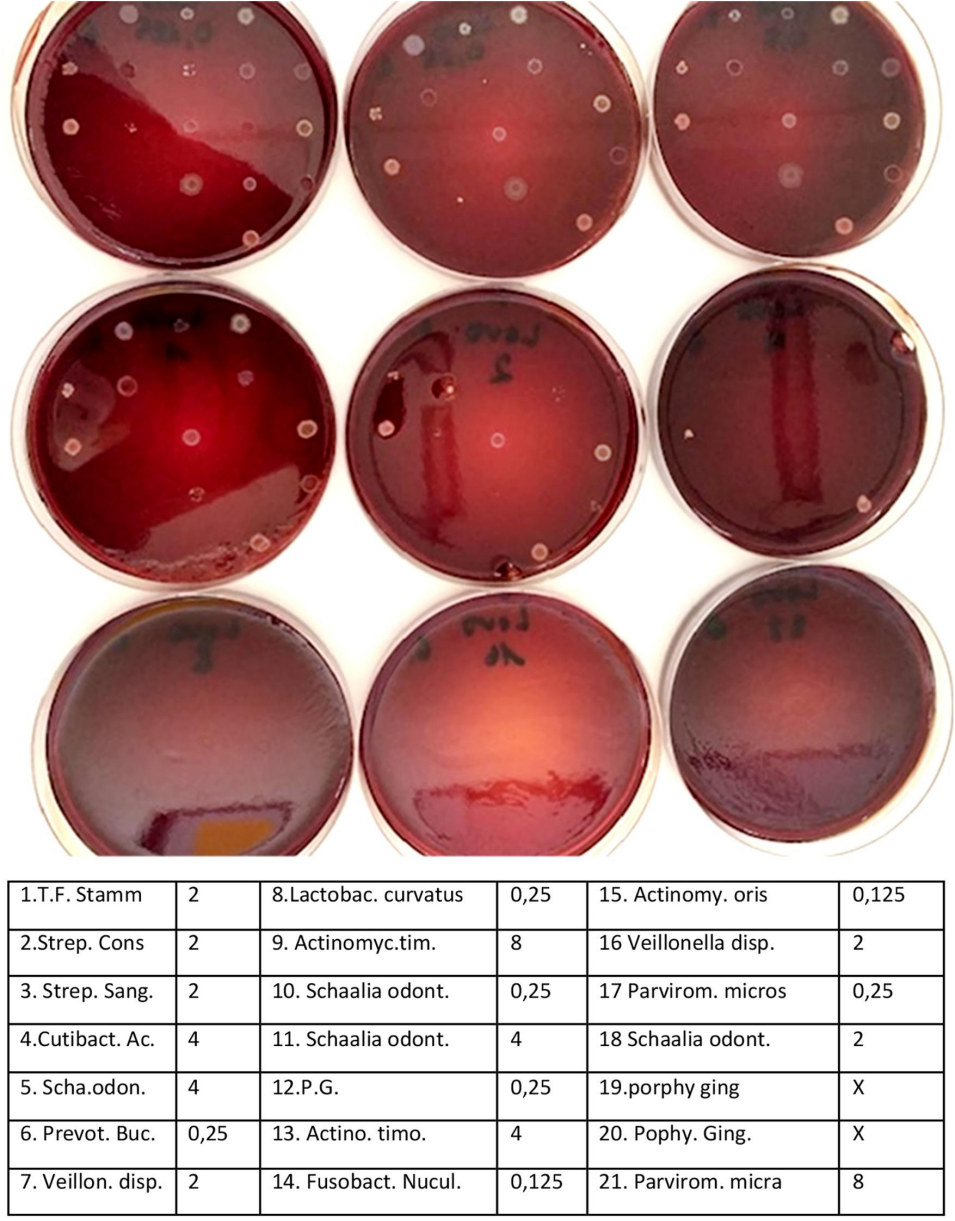
Documentation of results.

**Figure 3:**
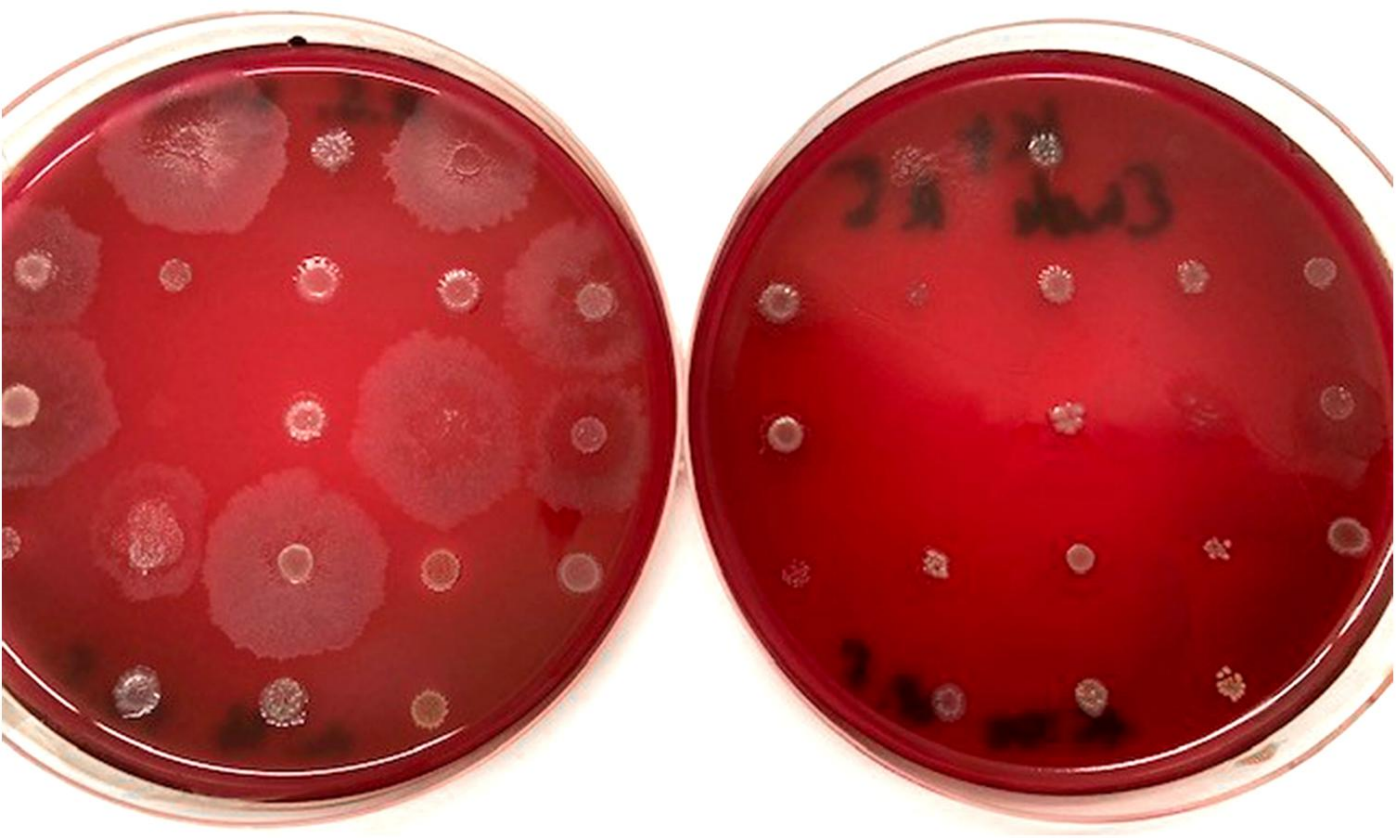
Control plates before each antibiotic series and after 2–3 min show viability of strains.

### 1.2 Antibiotics in periodontal disease

In severe periodontitis, antibiotics remain an essential adjunct to mechanical therapy; however, their misuse has increased antimicrobial resistance (AMR). Dentists are responsible for approximately 14% of all antibiotic prescriptions (15), and inappropriate prescribing is recognized by the World Health Organization as a major driver of AMR (16). Although β-lactams, macrolides, fluoroquinolones, and metronidazole remain key treatment options, their efficacy is increasingly limited. Responsible are resistance mechanisms, such as β-lactamase activity, efflux pumps, and porin modifications (17). Biofilm formation further complicates therapy, as microorganisms embedded in extracellular matrices can demonstrate resistance levels up to 100–1,000 times greater than those of planktonic cells (18). The positive interaction between *F. nucleatum* and T*. forsythia* is currently a widely studied phenomenon, for which there are also several explanatory models regarding the physiological and molecular biological relationships, including the following: In 2004, Sharma et all (19) reported, that cell to cell contact between *F.nucleatum* with *T.forsythia* are essential for the positive synergistic biofilm effect. In addition for forming a substratum for attachment *F. nuculatum* causes a suitable environment for *T.forsythia*. by generate reducing conditions which may be beneficial for strict anaerobes less tolerant of oxygen. 2017 Ruscitto et all (20) stated that *T. forsythia*, to overcome its inability to produce N-Acetlymuramic acid by itself can utilize muropeptides derived from *F. nucleatum* via transport through a muropeptide permease.

From a clinical perspective, rapid and reliable resistance testing of subgingival pathogens is beneficial to periodontal therapy. On a population level, the availability of an efficient, low-cost system for anaerobic dental pathogen testing contributes to rational antibiotic stewardship and sustainable resource use. In patients with recurrent infections unresponsive to previous antibiotic regimens, the application of this method can help identify the most effective drug. This might improve treatment success and prevent further systemic complications. Integrating this approach into routine dental practice can thus support individualized patient care and broader public health goals.

### 1.3 Multipoint inoculator in minimal inhibition concentration testing

A multipoint inoculator is an efficient equipment for parallel inoculation of multiple microbial isolates on semisolid media, significantly reducing the amount of work required and ensuring good reproducibility (21, 22). Since its introduction in the 1950s, it has been widely used in early antibiotic resistance research and in replica plating. However, it has not yet been integrated into routine clinical diagnostics due to technical issues. This might be for example limited surface coverage, high risk of cross-contamination, and labor-intensive handling (23). As automated antimicrobial susceptibility testing (AST) systems have not yet been validated for many obligate anaerobic bacteria, the multipoint inoculator is a reliable option for recording resistance profiles and trends, particularly in resource-limited laboratories (24).

The multipoint inoculator method offers a cost-effective and scalable approach to antibiotic susceptibility testing of anaerobic bacteria. Rapid and standardized transfer of multiple isolates to a single agar plate minimizes oxygen exposure, a critical factor in maintaining anaerobic viability and test reliability. Compared to E-test strips and commercially available anaerobic micro dilution plates—both of which are typically limited to testing a single strain, involve high costs per sample, and have a limited shelf life after opening and require high plastic waste—the multipoint inoculator allows up to 21 strains to be tested simultaneously within a short time (2 min), significantly increasing throughput. Unlike commercial systems, powder-based media components allow for long-term storage and preparation as needed, reducing material waste, storage requirements, and overall costs. Needles for inoculation can be sterilized and re-used. Although this is more labor-intensive than ready-to-use media, this trade-off is beneficial in settings where staff availability outweighs financial and infrastructure constraints.

This pilot study aimed to evaluate the methodological suitability of the multipoint inoculator for anaerobic oral pathogens and mixed biofilms. This should provide clinically relevant insights into resistance patterns that in addition enable antibiotic monitoring in dentistry. The method is particularly suitable for smaller laboratories, proof-of-concept studies, and dental clinics in low- and middle-income countries or remote regions where access to centralized microbiology laboratories is limited. The simultaneous incubation of multiple isolates on a single plate enables rapid visual detection of resistant subpopulations in patient samples, supporting timely, informed treatment decisions in decentralized clinical settings.

## 2 Materials and methods

### 2.1 Study design and sample collection

Mixed samples were obtained from routine subgingival plaque specimens collected from patients with chronic periodontitis at the University Dental Clinic Vienna. All patients exhibited periodontal pockets with a depth of 0.4–12 mm. The samples had been stored in strain collection at −20 °C. During processing, exposure to ambient air was limited to a maximum of 10 min to preserve viability of oxygen-sensitive species. No new human or animal sampling was performed. The Ethics Committee of the Medical University of Vienna (25 November 2020) confirmed that formal ethical approval was not required. The study used discarded, fully anonymized and irreversibly de-identified clinical samples; no patient-identifiable data were available.

### 2.2 Bacterial isolation and identification

Frozen isolates from stock samples were cultured on Müller–Hinton agar plates supplemented with 5% horse blood (Oxoid PB5303A). The cultures were incubated at 37 °C for 5–15 days in an anaerobic chamber (Whitley DG250 anaerobe workstation) in an environment with 80% N_2_, 15% CO_2_, and 5% H_2_. To rule out contamination, colonies are subjected to identification via matrix-assisted laser desorption/ionization time-of-flight mass spectrometry (MALDI-TOF), a widely established method for microbial diagnostics (25). Unclearly identified isolates were excluded. Isolate preparation: Strains were transferred to thioglycolate broth supplemented with vitamin K and hemin (BD BBL 221887) or to brain–heart infusion broth (BD BBL 220837) and cultured anaerobically at 37 °C for 48–72 h.

### 2.3 Antibiotic susceptibility testing

This pilot study included five antibiotics (amoxicillin ± clavulanic acid, imipenem, clarithromycin, and levofloxacin) from different pharmacological classes (Table 1). Amoxicillin and amoxicillin/clavulanate were included as established first-line β-lactams in odontogenic and periodontal infections. Imipenem was tested as a broad-spectrum carbapenem representing a reserve (“last-line”) β-lactam. Levofloxacin was selected due to previously reported variable *in vitro* activity against anaerobic bacteria (26). Clarithromycin was included as it has been investigated as an adjunct to non-surgical periodontal therapy with evidence suggesting potential clinical benefits in selected settings, supporting its evaluation in the present study (27).

**Table 1:** Antibiotics used for testing.

Antibiotic class	Antibiotic (catalog number)	Source
β-lactam	Amoxicillin (A8523)	Sigma
β-lactam + inhibitor	Amoxicillin + clavulanic acid (SMB00607)	Sigma
Carbapenem	Imipenem (1009000)	Pharmacopoeia
Macrolide	Clarithromycin (C9742)	Sigma
Quinolone	Levofloxacin (28266)	Sigma

### 2.4 Inoculation procedure

Preliminary findings indicated that certain anaerobes only grow stably and reproducibly at higher cell densities. This is consistent with established anaerobic methodologies, as EUCAST disk diffusion testing for anaerobes (Version 13.1.) is also standardized at McFarland 1.0 (28). Bacterial suspensions were standardized to 1 McFarland (∼3 × 10^8^ CFU/mL) in Mueller–Hinton broth (AppliChem 413788). A multipoint inoculator (Denley A400) was used to perform inoculation. Inoculation of 1–2 µL bacterial suspensions onto pre-incubated antibiotic plates. Plates (FAA-Ager W1761 Millipore+5%Horseblood SR0050D Thermo) contain antibiotic concentrations between 0.125 mg/L and 32 mg/L and are prepared up to five days before the multipoint inoculation test is performed. They are stored at 8 °C and pre-incubated in the anaerobic chamber 6 h before inoculation. The inoculation per antibiotic series takes 2–3 min per series (10 plates). The plates were immediately transferred to the anaerobic chamber (Whitley DG250 anaerobe workstation) and incubated at 37 °C for 72 h up to 7 days, depending on the growth. An antibiotic-free control plate was included in each run.

MIC breakpoints for amoxicillin, amoxicillin/clavulanate, imipenem, and levofloxacin (with moxifloxacin used as a surrogate for fluoroquinolones) were interpreted according to **EUCAST** guidelines for anaerobes (28) and guidelines [“When there are no breakpoints”, version 30 June 2023, (Table 2)] (30). For clarithromycin, the Lorian table (Table 2.12): “Zone diameters and equivalent MIC breakpoints for organisms other than *Haemophilus*, *Neisseria gonorrhoeae*, and *Streptococcus pneumoniae*” (30) was applied. Antimicrobial susceptibility testing results were used to calculate the proportion of isolates with elevated MIC values according to the applied interpretive criteria and to determine MIC50 and MIC90 values for each antimicrobial agent. Only isolates showing clearly interpretable growth inhibition were included in the analysis.

**Table 2:** Comparison alternative AST systems.

Aspect	Multipoint inoculator	E-test	Broth micro dilution
Throughput	High (21 strains/plate)	Low (1 strain/strip)	Moderate (1 strain/panel)
Cost per isolate	Low	High	Moderate–high
Labor effort	Moderate manual work	low	low (commercial panels)
Avoid Oxygenstress	Very fast processing	Depending on experience	Depending on experience
Environmental impact	Low (reusable, autoclavable inoculation pins),	Moderate (single-use strips,	High (single-use plastic plates/ panels, tips)
Main limitations	Cross-contamination risk; manual work	Expensive; single-use	cost; specialized materials
Typical use	Research, screening, resource-limited labs	Individual MIC testing	Clinical routine AST

### 2.5 Controls and additional experiments

- Oxygen stress controls: To evaluate bacterial loss during handling, control plates were prepared before and after inoculation to confirm minimal viability loss within a 30 min preparation window.
- Mixed-inoculum assay: Reference strains (*P. gingivalis* ATCC 33277, *T. forsythia* ATCC 43037, *F. nucleatum* OMZ598) were tested individually and in combination. As *T. forsythia* requires external N-acetylmuramic acid, 20 µg/mL was added to the blood agar (FAA + Horseblood).

## 3 Results

### 3.1 General observations

Influence of time (oxygen stress): Inoculation typically requires approximately 30 min to finish; however, inoculation performed using the multipoint inoculator took only 2–3 min exposure. The applied conditions and media supported consistent bacterial growth and enabled reliable determination of minimum inhibitory concentrations (MICs) no. per antibiotic series (nine concentrations plus control). Control plates prepared before and after each inoculation run exhibited minimal or no bacterial losses due to air. From an initial set of 30 isolates per species or genus (35 for *Porphyromonas gingivalis*), the number of successfully identified and evaluable isolates is reflected in the reported N values for each organism.

Following cultivation prior to antimicrobial susceptibility testing, several isolates were excluded due to identification difficulties using MALDI-TOF MS. Specifically, 5 dark-pigmented *P. gingivalis* isolates and 9 dark-pigmented *Prevotella* spp. could not be reliably identified. In addition, identification failed for 8 *Parvimonas micra* isolates, 5 *Actinomyces* spp. isolates, and 8 *Veillonella* spp. isolates.The difference between the initial number of isolates subjected to multipoint inoculation and the final number of evaluable isolates represents losses occurring during multipoint inoculation and subsequent incubation. These included the loss of 4 *Porphyromonas* spp., 7 *Prevotella* spp., 7 *Actinomyces* spp., 7 *Parvimonas micra*, and 7 *Veillonella* spp. isolates, most likely due to contamination or insufficiently interpretable growth inhibition.

#### 3.1.1 *Porphyromonas* spp.

**Figure 4:**
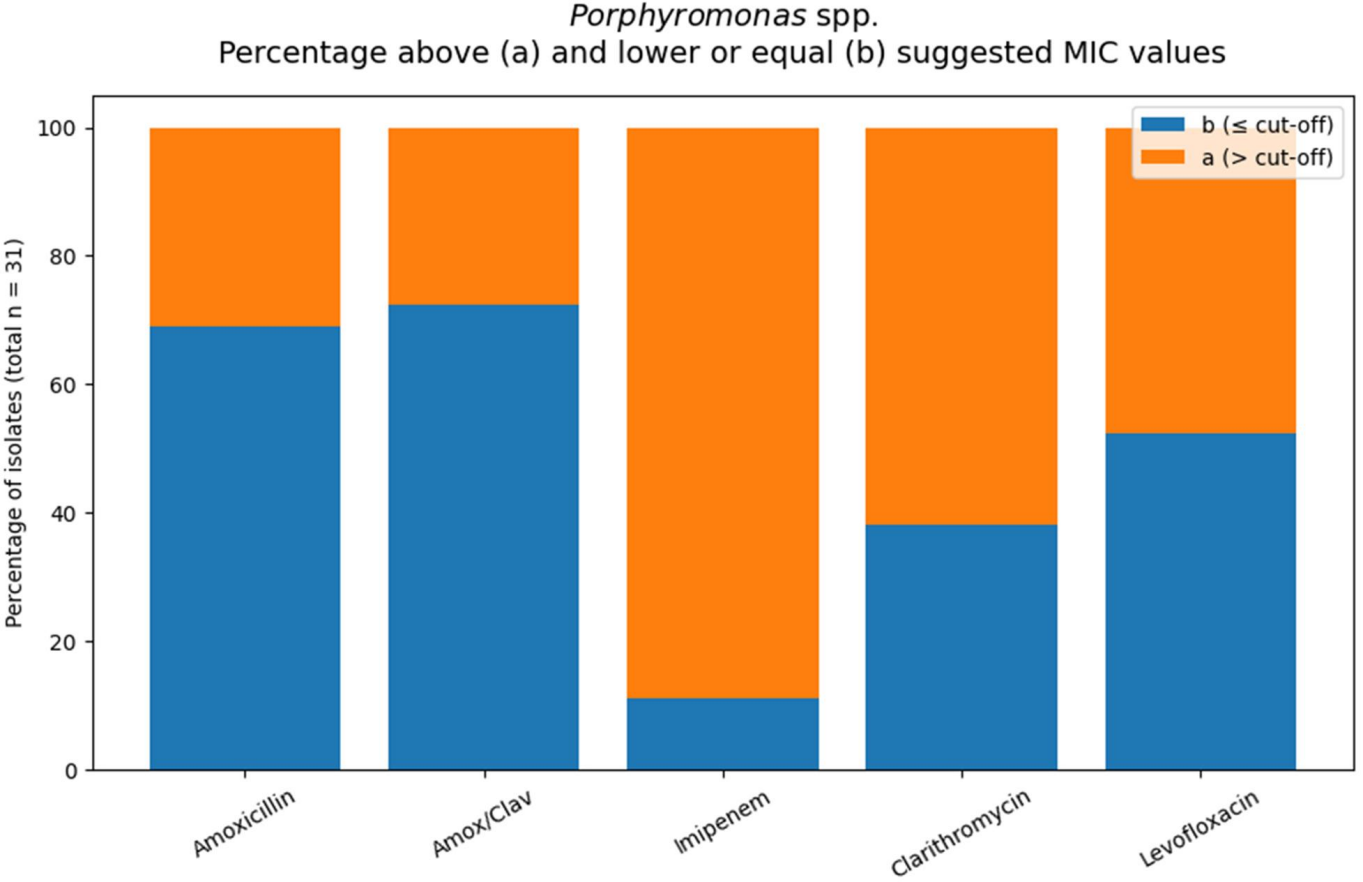
Orange areas indicate isolates with MIC values above the applied interpretive threshold for the respective antimicrobial agents (total number of tested *Porphyromonas* spp. isolates: *n* = 31).

Based on the suggested MIC orientation values, 31% of isolates exceeded the value for amoxicillin and 28% for amoxicillin/clavulanate. For levofloxacin and clarithromycin, 48% and 62% of isolates, respectively, were categorized as (a) (Table 3; Figure 4).

**Table 3:** *Porphyromonas* spp, MIC values according to the applied interpretive criteria.

Antibiotic	MIC sug. (mg/L)	*N*	MIC50	MIC90	*N*^b^ (≤cut-off)	*N*^a^ (>cut-off)	%^a^
Amoxicillin	0.5	29	0.125	2	20	9	31%
Amox/Clav	0.5	29	0.25	2	21	8	28%
Imipenem	1	27	>32	>32	3	24	89%
Clarithromycin	0.5	21	4	>32	8	13	62%
Levofloxacin	1	21	1	4	11	10	48%

MIC:Amox, Amox/Clav, Imipenem and Levofloxacin (Moxifloxacin) from Eucast “When there are no breakpoints”, 30.06.2023, (Table 2) (30)”; Clarythromycin, Lorian (Table 1.12) (30).

^a^ “Formal categorizing of the susceptibility of the organism is not possible. The MIC suggest that the agent should not be used for therapy”.

^b^ “Formal categorizing the susceptibility of the organism not possible. A caution interpretation suggests that the agent may be considered for therapy”.

Imipenem MICs frequently lay above the suggested threshold with all tested antibiotics; this finding is interpreted as a technical problem and addressed in the discussion.

Overall, susceptibility appeared agent-dependent, with no consistently high activity across all tested drug classes.

#### 3.1.2 *Prevotella* spp.

**Figure 5:**
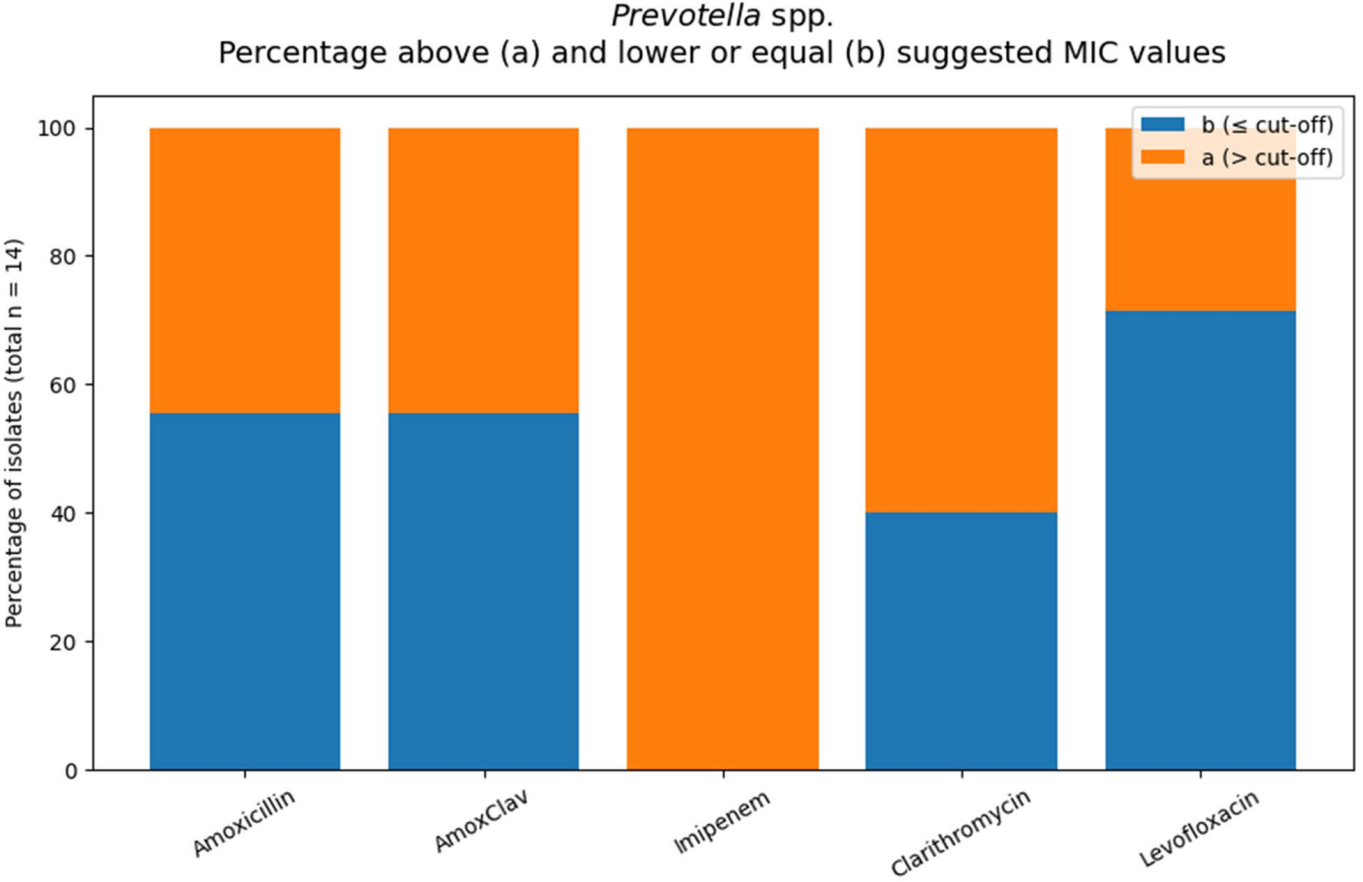
Orange areas indicate isolates with MIC values above the applied interpretive threshold for the respective antimicrobial agents (total number of tested *Prevotella* spp. isolates: *n* = 14).

Using the Breakpoint Tables and guidelines (28–30) for orientation values, 44% of isolates exceeded the value for amoxicillin and amoxicillin/clavulanate. (Table 4; Figure 5) Suggested MIC levofloxacin, 29% of isolates were categorized as (a), while clarithromycin (MIC due to Lorian) showed higher proportions (60%). MIC distributions varied across antimicrobial agents. MIC_50_/MIC_90_ values were 0.25/2 mg/L for amoxicillin and 0.25/1 mg/L for amoxicillin/clavulanate, indicating predominantly low MICs with some higher values. Levofloxacin showed a broader spread (MIC_50_ 0.25 mg/L; MIC_90_ 2 mg/L). In contrast, clarithromycin demonstrated higher MIC levels overall (MIC_50_ 2 mg/L; MIC_90_ > 32 mg/L).

**Table 4:** *Prevotella* spp, MIC values according to the applied interpretive criteria.

Antibiotic	MIC sug. (mg/L)	*N*	MIC50	MIC90	N^b^ (≤cut-off)	*N*^a^ (>cut-off)	%^a^
Amoxicillin	0.25	9	0.25	2	5	4	44%
Amox/Clav	0.25	9	0.25	1	5	4	44%
Imipenem	0.125	12	>32	>32	0	12	100%
Levofloxacin	1	7	0.25	2	5	2	29%
Clarithromycin	0.5	10	2	>32	4	6	60%

^a^ “Formal categorizing of the susceptibility of the organism is not possible. The MIC suggest that the agent should not be used for therapy”.

^b^ “Formal categorizing the susceptibility of the organism not possible. A caution interpretation suggests that the agent may be considered for therapy”.

#### 3.1.3 *Actinomycetes* spp.

**Figure 6:**
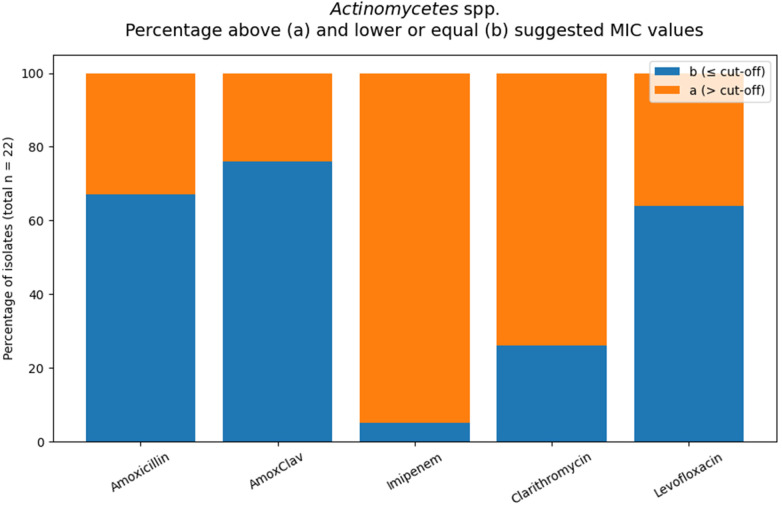
Orange areas indicate isolates with MIC values above the applied interpretive threshold for the respective antimicrobial agents (total number of tested Actinomycetes spp. isolates: *n* = 22.

Suggested MIC orientation values indicates 33% of isolates exceeded the value for amoxicillin and 24% for amoxicillin/clavulanate. For levofloxacin and clarithromycin, 36% and 74% of isolates, respectively, were categorized as (a) (Figure 6).

MIC distributions showed agent-dependent variability. MIC_50_/MIC_90_ values were 0.125/2 mg/L for amoxicillin and 0.25/1 mg/L for amoxicillin/clavulanate, indicating generally low MICs with some higher values. Levofloxacin demonstrated a broader distribution (MIC_50_ 0.25 mg/L; MIC_90_ 2 mg/L). Clarithromycin showed elevated MIC levels overall (MIC_50_ 4 mg/L; MIC_90_ > 32 mg/L) (Table 5).

**Table 5:** *Actinomycetes* spp. MIC values according to the applied interpretive criteria.

Antibiotic	MIC sug.	*N*	MIC50	MIC90	*N*^b^ (≤cut-off)	*N*^a^ (>cut-off)	%^a^
Amoxicillin	0.5	21	0.125	2	14	7	33%
Amox/Clav	0.5	21	0.25	1	16	5	24%
Imipenem	1	22	>32	>32	1	21	95%
Clarithromycin	0.5	19	4	>32	5	14	74%
Levofloxacin	1	11	0.25	2	7	4	36%

MIC: Amox, Amox/Clav, Imipenem and Levofloxacin (Moxifloxacin) from Eucast “When there are no breakpoints”, 30.06.2023, (Table 2) (30)”; Clarythromycin, Lorian (Table 1.12) (30).

^a^ “Formal categorizing of the susceptibility of the organism is not possible. The MIC suggest that the agent should not be used for therapy”.

^b^ “Formal categorizing the susceptibility of the organism not possible. A caution interpretation suggests that the agent may be considered for therapy”.

#### 3.1.4 *Veillonella* spp.

**Figure 7:**
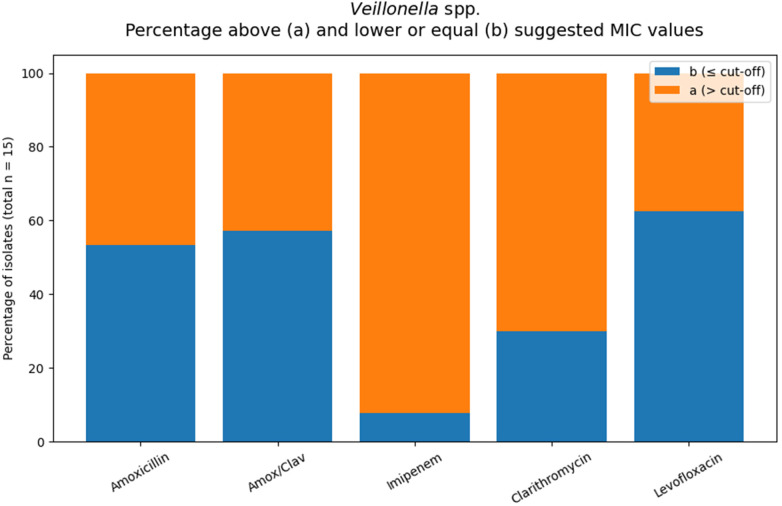
Orange areas indicate isolates with MIC values above the applied interpretive threshold for the respective antimicrobial agents (total number of tested *veillonellas* spp. isolates: *n* = 15.

Based on the suggested MIC orientation values, 47% of isolates exceeded the value for amoxicillin and 43% for amoxicillin/clavulanate. For levofloxacin and clarithromycin, 38% and 70% of isolates, respectively, were categorized as (a)MIC values varied between antimicrobial agents (Table 6; Figure 7). MIC_50_/MIC_90_ values were 0.5/4 mg/L for amoxicillin and amoxicillin/clavulanate, indicating a distribution extending into higher MIC ranges. Levofloxacin showed MIC_50_ and MIC_90_ values of 0.5 and 2 mg/L, respectively. Clarithromycin demonstrated generally elevated MIC levels (MIC_50_ 2 mg/L; MIC_90_ > 32 mg/L (Table 6).

**Table 6:** *Veillonella* spp. MIC values according to the applied interpretive criteria.

Antibiotic	MIC sug.	*N*	MIC50	MIC90	*N*^b^ (≤cut-off	*N*^a^ (>cut-off)	%^a^
Amoxicillin	0.5	15	0.5	4	8	7	46.7%
Amox/Clav	0.5	14	0.5	4	8	6	42.9%
Imipenem	1	13	>32	>32	1	12	92.3%
Clarithromycin	0.5	10	2	>32	3	7	70%
Levofloxacin	1	8	0.5	2	5	3	37.5%

MIC: Amox, Amox/Clav, Imipenem and Levofloxacin (Moxifloxacin) from Eucast “When there are no breakpoints”, 30.06.2023, Table 2 (30)”; Clarythromycin, Lorian Table 1.12 (30).

^a^ “Formal categorizing of the susceptibility of the organism is not possible. The MIC suggest that the agent should not be used for therapy”.

^b^ “Formal categorizing the susceptibility of the organism not possible. A caution interpretation suggests that the agent may be considered for therapy”.

#### 3.1.5 Parvimonas micra

**Figure 8:**
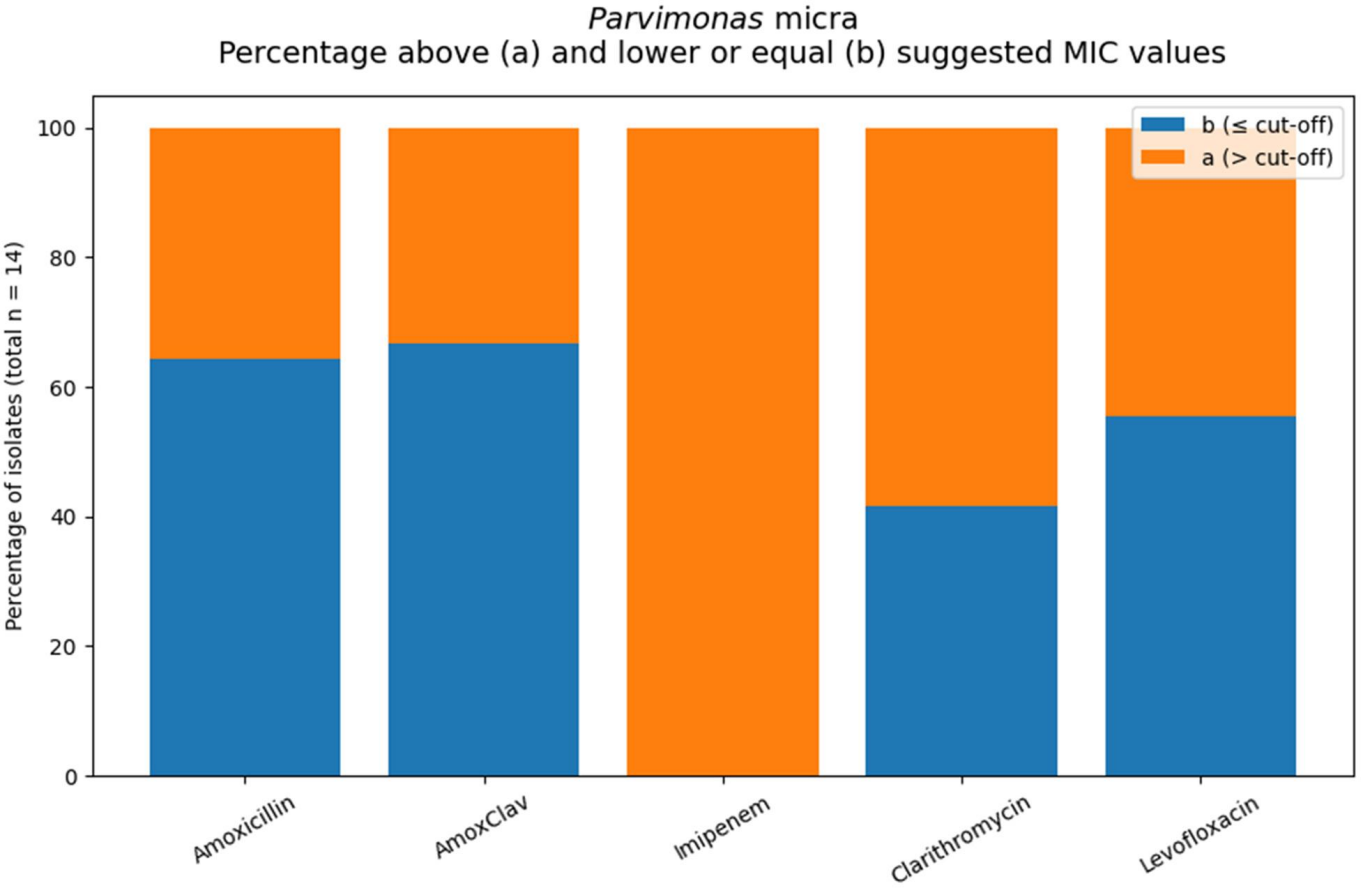
Orange areas indicate isolates with MIC values above the applied interpretive threshold for the respective antimicrobial agents (total number of tested *Parvimonas micra*. isolates: *n* = 14.

Among *Parvimonas micra* isolates, susceptibility varied by agent. Moderate proportions exceeded the suggested MIC values for amoxicillin (35.7%) and amoxicillin/clavulanate (33.3%), while higher rates were observed for levofloxacin (44.4%) and clarithromycin (58.3%). MIC₅₀/MIC₉₀ values were 0.25/4 mg/L for amoxicillin and amoxicillin/clavulanate, 0.5/8 mg/L for levofloxacin, 2/>32 mg/L for clarithromycin. (Table 7; Figure 8).

**Table 7:** *Parvimonas micra*. MIC values according to the applied interpretive criteria.

Antibiotic	MIC sug.	*N*	MIC50	MIC90	*N*^b^ (≤cut-off)	*N*^a^ (>cut-off)	%^a^
Amoxicillin	0.5	14	0.25	4	9	5	35.7%
Amox/Clav	0.5	15	0.25	4	10	5	33.3%
Imipenem	1	13	>32	>32	0	13	100%
Clarithromycin	0.5	12	2	>32	5	**7**	58.3%
Levofloxacin	1	9	0.5	8	5	4	44.4%

MIC: Amox, Amox/Clav, Imipenem and Levofloxacin (Moxifloxacin) from Eucast “When there are no breakpoints”, 30.06.2023, Table 2 (29)”; Clarythromycin, Lorian Table 1.12 (30).

^a^ “Formal categorizing of the susceptibility of the organism is not possible. The MIC suggest that the agent should not be used for therapy”.

^b^ “Formal categorizing the susceptibility of the organism not possible. A caution interpretation suggests that the agent may be considered for therapy”.

### 3.2 Results – gram-positive vs. gram-negative comparison

**Figure 9:**
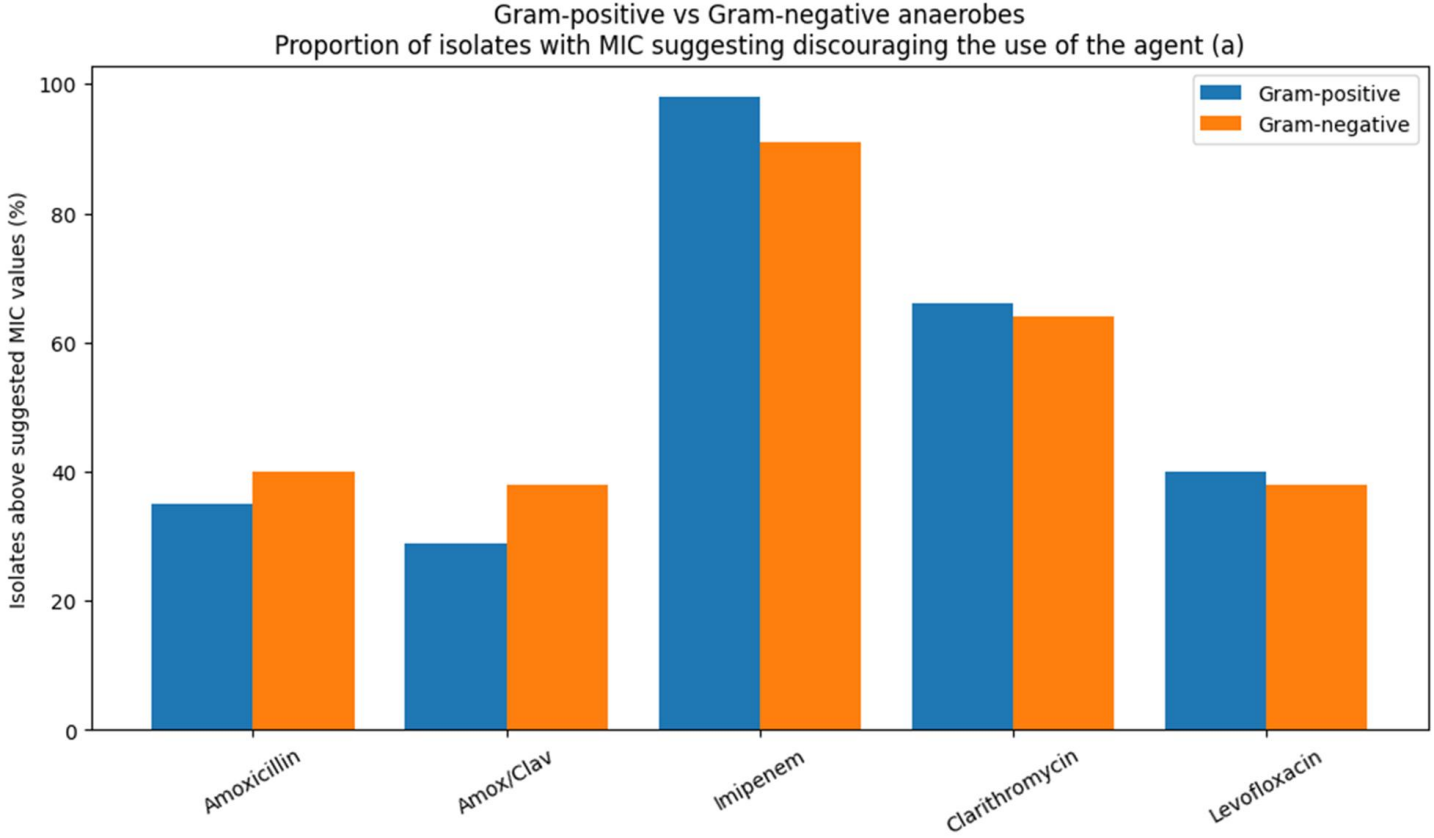
Comparison of gram-positive and gram-negative anaerobic bacteria regarding the proportion of isolates with MIC values exceeding the suggested MIC orientation values (category a). Gram-positive organisms: *Parvimonas micra* and *Actinomycetes* spp., gram-negative organisms: *Porphyromonas* spp., *Prevotella* spp., and *Veillonella* spp.

When grouped by Gram reaction (Table 8; Figure 9), no pronounced overall difference in susceptibility patterns was observed between Gram-positive (*Parvimonas micra*, *Actinomycetes* spp.) and Gram-negative anaerobes (*Porphyromonas* spp., *Prevotella* spp., *Veillonella* spp.). Instead, the proportion of isolates exceeding the suggested MIC orientation values appeared primarily agent-dependent.

**Table 8:** Gram-positive/Gram-negative.

Group	Spezies/Genera	Amox	Amox/Clav	Imipenem	Clarithro	Levoflox
Gram-positiv	*Actinomycetes* spp.	26%	22%	95%	37%	28%
	*Parvimonas micra*	50%	36%	100%	38%	33%
Gram-negativ	*Porphyromonas* spp.	31%	28%	89%	48%	15%
	*Prevotella* spp.	36%	40%	100%	45%	0%
	*Veillonella* spp.	47%	38%	88%	36%	50%

For amoxicillin and amoxicillin/clavulanate, moderate proportions of isolates were categorized as (a) in both groups, with slightly higher values among Gram-negative species. Clarithromycin showed elevated proportions of (a) isolates across both Gram-positive and Gram-negative bacteria, indicating reduced *in vitro* activity irrespective of Gram classification. Levofloxacin demonstrated intermediate values without a consistent Gram-related trend. Imipenem MICs frequently exceeded the suggested orientation threshold in both groups; this observation is interpreted cautiously and is addressed to technical issues in the discussion. Overall, susceptibility patterns were not strongly associated with Gram reaction but were mainly determined by the individual antimicrobial agent (Figure 9).

### 3.3 Mixed-inoculum assay

**Figure 10:**
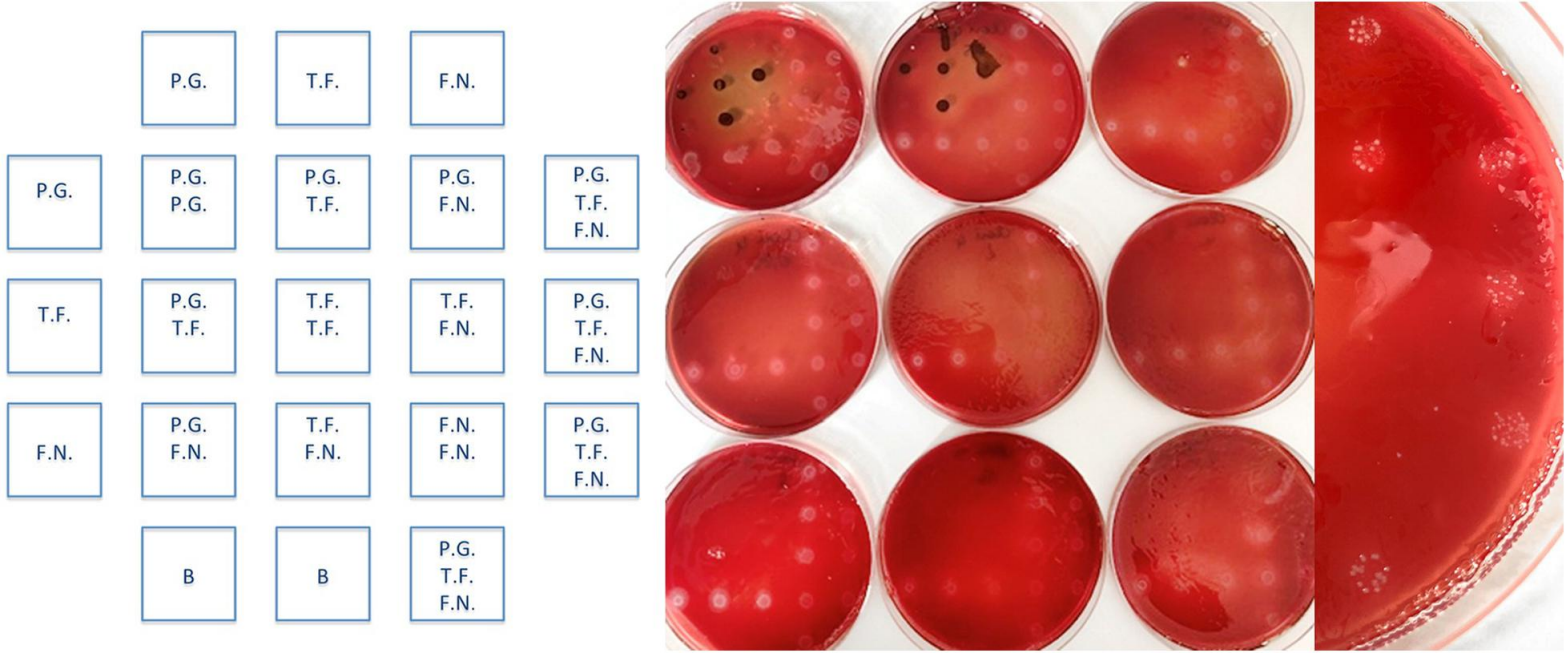
The left panel shows the reference inoculation layout used to assign individual *P. gingivalis, T. forsythia*, and *F. nucleatum* isolates to the matched inocula in the center and right panels. In the mixed inoculum (far left), two isolates showed visible growth, indicating no growth inhibition, even at the highest tested concentrations of amoxicillin and amoxicillin/clavulanate (32 mg/L).

In the upper and left rows (Figure 10), *P. gingivalis* (P.G.), *T. forsythia* (T.F.), and *F. nucleatum* (F.N.) were separately inoculated and then combined like in the schedule on the right side. Figure 10 *T. forsythia* exhibited increased tolerance to amoxicillin when cultured with *F. nucleatum* (third row from the top and left). Although individual strains stopped growing at 0.125–0.5 mg/L, *T. forsythia* persisted in combination at concentrations of up to 32 mg/L for Amoxicillin and Amoxicilin/Clavulan, indicating the influence of microbial interactions on resistance. The last row on the right, in which all three bacteria were inoculated together, also shows the growth of *T. forsythia* and F. nucleatum but not *P. gingivalis*. B in the last row stands for control broth (contamination control).

## 4 Discussion

As a reminder, this was a pilot study. This pilot evaluated the feasibility of using a multipoint inoculator for antimicrobial susceptibility testing of anaerobic oral pathogens. Although the equipment yielded positive outcomes, several limitations must be acknowledged. The cultivation of strict anaerobes posed challenges, particularly due to prior storage at −20 °C, which may have reduced bacterial viability and therefore to loss during procedure. Future studies should adopt cryopreservation at −80°C, lyophilization (31), or storage in liquid nitrogen (32) to preserve anaerobic strains. Furthermore, slow-growing species, such as *P. gingivalis* and *T. forsythia*, complicate the identification by MALDI-TOF. MALDI-TOF, although rapid and practical, has limitations for dark-pigmented anaerobes (25). Some isolates yielded insufficient biomass for an accurate analysis (*Parvimonas micra*). Therefore, complementary molecular methods, such as MicroIDent PCR, may be required, although these cannot distinguish between viable and nonviable organisms (33). Moreover, the relatively small number of isolates per species limits the statistical power and prevents definitive conclusions regarding resistance prevalence. A larger follow-up study involving at least 50–60 isolates per species is warranted to establish species-specific MIC distributions and reliable breakpoints.

Compared with traditional susceptibility testing, the use of the multipoint inoculator provided several methodological benefits. First, simultaneous inoculation of up to 21 isolates per plate shortened the handling time outside the anaerobic chamber and reduced oxygen stress, which is particularly crucial for highly sensitive species, such as *P. gingivalis* and *T. forsythia*. Second, the equipment enabled co-inoculation of multiple species, and our mixed-culture experiment revealed increased tolerance of *T. forsythia* when co-cultured with *F. nucleatum*, highlighting the clinical importance of interspecies interactions in biofilms. Sharma et al. 2004 (19) reported, that cell to cell contact between *F.nucleatum* with *T. forsythia* is essential for the positive synergistic biofilm effect. In addition for forming a substratum for attachment, *F. nuculatum* causes a suitable environment for *T. forsythia*. by generate reducing conditions which may be beneficial for strict anaerobes less tolerant of oxygen. 2017 Ruscitto et all (20) stated that *T. forsythia*, to overcome its inability to produce N-Acetlymuramic acid byitself can utilize muropeptides derived from *F. nucleatum* via transport through a muropeptide permease (TfAmpG) whose expression is regulated by a unique hybrid two-component system (Tf GppX). Combining the multi-species-multipoint inoculation protocol with sequencing of muropeptide expression at multiple stages during and after inoculation could therefore be a next step toward gaining deeper insights into the changes in antimicrobial susceptibility associated with the cell wall and cell membrane. Third, the approach reduced costs by approximately 70%–80% per antibiotic–isolate combination compared with E-tests or broth microdilution. E-strips require one plate and one strip per isolate, whereas the multipoint approach uses 10 plates per antibiotic for 21 isolates (nine concentrations plus one control), resulting in markedly lower total costs at a higher throughput. Collectively, these advantages support the use of the multipoint inoculator as a practical, scalable, and ecologically favorable equipment in research and diagnosis.

Across all investigated anaerobic taxa, susceptibility patterns were mainly agent-specific rather than determined by Gram classification, and no consistent advantage was observed for Gram-positive or Gram-negative organisms.

Amoxicillin and amoxicillin/clavulanate showed generally moderate MIC_50_ values but with a relevant subset of higher MICs, resulting in moderate proportions of isolates exceeding the suggested orientation values. Clarithromycin displayed consistently elevated MIC distributions and high proportions of category a (discouraging the use of agent) isolates across taxa, indicating limited *in vitro* suitability though clinical studies have reported potential benefits as an adjunct in periodontal therapy (27), highlighting a discrepancy between laboratory susceptibility data and observed clinical effects. Levofloxacin showed intermediate activity without a clear Gram-related trend.

Overall, antimicrobial activity against oral anaerobes appeared strongly drug-dependent, and neither Gram classification nor taxonomic grouping reliably predicted susceptibility patterns.

The high resistance rates observed to imipenem (75%–100%) should be interpreted with caution and do not reflect the actual clinical resistance situation. Rather, these results primarily highlight technical challenges in handling imipenem in *in vitro* susceptibility testing. As a carbapenem used as a reserve antibiotic in severe systemic infections, reliable determination of activity is particularly important. However, the known instability of imipenem (34) represents a significant methodological limitation. Rapid hydrolysis and temperature-dependent degradation can significantly reduce bactericidal activity over time, leading to an overestimation of resistance rates (34, 35). The plates were stored between two to five days. The present results therefore serve primarily to draw attention to the importance of stability, storage conditions, and handling when using imipenem in experimental approaches in order to avoid misinterpretations and to raise awareness of this methodology for future applications. Overall, the highest resistance rates were observed beside imipenem towards clarithromycin, while lower resistance rates were found for amoxicillin (with or without clavulanic acid) and levofloxacin.

### 4.1 Clinical relevance and conclusion

These findings argue against the empirical use of macrolides or carbapenem in dental infections, whereas amoxicillin and, in selected cases with infections caused by anaerobic gram-negative oral pathogens, levofloxacin, remain the most reliable options, out of the tested Ab-spectrum, pending susceptibility testing. We explicitly emphasize that the observations described herein are based solely on *in-vitro* findings and do not constitute any form of therapeutic recommendation. Treatment decisions must be made exclusively by qualified medical professionals and tailored to each individual case.

These resistance patterns have clinical implications that extend beyond the oral cavity, as periodontal pathogens can systemically disseminate and are associated to certain conditions, such as infective endocarditis, Alzheimer's disease, colorectal cancer, and chronic inflammatory disorders, including diabetes mellitus, inflammatory bowel disease, and cardiovascular disease (2, 36). Untreated infections increase healthcare costs and reduce life expectancy. As dentists prescribe approximately 14% of all antibiotics (15), rational antimicrobial stewardship in dentistry is crucial. The present study shows that the multipoint inoculator can provide clinically relevant data for dentistry on two complementary levels. At the population level, a reliable, economical, and ecologically favorable system for resistance testing in anaerobic oral pathogens enhances antimicrobial stewardship. By enabling surveillance of resistance trends with minimal resources, this approach supports evidence-based prescribing and reduces unnecessary use of broad-spectrum or last-resort antibiotics, thereby supporting public health. The resistance rates observed highlight the urgent need for ongoing surveillance and rational antibiotic use in dentistry. Thus, the multipoint inoculator is not only a research equipment but also a potential adjunct to periodontal therapy, bridging the gap between laboratory diagnostics and clinical decision-making.

### 4.2 Outlook

Future studies should increase the number of isolates and rethink storage, broaden the antibiotic spectrum, and evaluate innovative adjuvant strategies to enhance antibiotic efficacy and support sustainable antimicrobial stewardship. The close proximity of clinical and analytical facilities offers the University Dental Clinic a distinct advantage in the analysis of fastidious anaerobes. Ongoing efforts focus on the development of a transport medium that preserves anaerobic gram-negative bacteria for reliable resistance monitoring of external samples. As resistance may differ in biofilm-associated bacteria compared with planktonic cultures, mixed inoculum and biofilm models will be systematically integrated.

## Data Availability

The raw data supporting the conclusions of this article will be made available by the authors, without undue reservation.
